# Association between giant cell glioblastoma and glioblastoma multiforme in the United States: A retrospective cohort study

**DOI:** 10.1002/brb3.1402

**Published:** 2019-08-29

**Authors:** Amro K. Bin Abdulrahman, Khalid A. Bin Abdulrahman, Yousef R. Bukhari, Abdulaziz M. Faqihi, Juan Gabriel Ruiz

**Affiliations:** ^1^ College of Medicine Imam Mohammad Ibn Saud Islamic University Riyadh Saudi Arabia; ^2^ Herbert Wertheim College of Medicine Florida International University Miami FL USA

**Keywords:** brain cancer, giant cell glioblastoma, glioblastoma multiforme, mortality, prognosis

## Abstract

**Objectives:**

The current study aims to find the differences between glioblastoma multiforme (GBM) and giant cell glioblastoma (GCG) regarding mortality and prognosis among adults and elderly patients in the U.S.

**Methods and Materials:**

This study is a historical cohort type of study and is conducted on adults and elderly individuals with GBM or GCG from the years 1985–2014 in the U.S. Data were collected from the Surveillance, Epidemiology, and End Results Program (SEER) database. The study exposure was GBM or GCG and the outcome was mortality. The potential confounders were age, sex, race, ethnicity, year of diagnosis, primary site, brain overlap, and surgery. A chi‐square test was used for categorical data. A univariate analysis was used for variables having a *p*‐value <.05. Potential confounders were selected and evaluated using multivariate logistic regression models to calculate the odds ratio with stepwise selection.

**Results:**

The study sample was 25,117. The incidences of GBM and GCG were not similar in relation to age group. Also, Spanish–Hispanic ethnicity was independently protective of GBM and GCG as compared to Non‐Spanish–Hispanic ethnicity patients with GBM have a higher mortality rate than do GCG patients. The mortality rate was higher among patients diagnosed before 2010.

**Conclusion:**

GCG was not statistically significant in association to reduced mortality. Non‐Spanish–Hispanics with GBM or GCG had a higher mortality rate than did Spanish–Hispanics. Factors such as being female, being age 59–65, and having a year of diagnosis before 2010 were independently associated with increased mortality.

## Graphical abstract

When comparing glioblastoma multiforme (GBM) and giant cell glioblastoma (GCG) using SEER database, the researches have found the following: GCG was not statistically significant in association with reduced mortality. Non‐Spanish–Hispanics with GBM or GCG had a higher mortality rate than did Spanish–Hispanics. Factors such as being female, being age 59–65, and having a year of diagnosis before 2010 were independently associated with increased mortality.

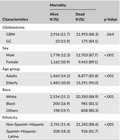

## 1 INTRODUCTION

Brain cancer and other nervous system cancers are the tenth leading cause of death in the U.S. Brain cancer is common among adults and elderly individuals (SEER Program). Giant cell glioblastoma (GCG) is a rare neoplasm characterized by a predominance of bizarre multinucleated giant cells with abundant eosinophilic cytoplasm. (Ohgaki, Peraud, Nakazato, Watanabe, & Deimling, 2000).

Significant efforts to characterize this unusual malignancy have established a glial origin, and it is now considered a subtype of glioblastoma multiforme (GBM; Akslen, Mork, Larsen, & Myrseth, 1988; Becker, Benyo, & Roessmann, 1967; Hadfield & Silverberg, 1972; Katoh et al., 1995; Kawano et al., 1995; Margetts & Kalyan‐Raman, 1989).

GCG has been reported to represent between 2% and 5% of GBM cases (Artico, Cervoni, Celli, Salvati, & Palma, 1993; Palma, Celli, Maleci, Di Lorenzo, & Cantore, 1989; Shinojima et al., 2004).

Importantly, several small series and case reports have suggested that the prognosis of GCG is significantly better than that observed for GBM (Akslen et al., 1988; Becker et al., 1967; Burger & Vollmer, 1980; Chang, Kuwana, Ito, Koike, & Kitamura, 2001; Deb, Sharma, Chander, Mahapatra, & Sarkar, 2006; Gullotta, Casentini, & Neumann, 1980; Klein, Molenkamp, Sorensen, & Roggendorf, 1998; Kroh, Matyja, Marchel, & Bojarski, 2004; Margetts & Kalyan‐Raman, 1989; Sabel, Reifenberger, Weber, Reifenberger, & Schmitt, 2001; Shinojima et al., 2004). The development of GBG is highly related to mutations of the *TP53* gene (Kleihues & Glioblastoma,)

*MGMT* promoter methylation, mutations in the *IDH1/2* genes, or *BRAF* mutations, which are actually used as diagnostic, prognostic, and predictive molecular markers in anaplastic glial tumors (Lohkamp et al., 2016).

Glioblastoma multiforme is a common malignant tumor that originates from astrocytes. It is a rapid‐growing tumor that affects the nervous system, including the brain and the spinal cord (GBM, 2017).

It is estimated that GBM cases in the U.S. account for approximately 20% of all primary CNS tumors in the adult population and almost 75% of all anaplastic gliomas (Nizamutdinov et al., 2018). Glioblastoma multiforme is the most lethal primary malignant central nervous system tumor in adults (Li et al., 2015; Ohgaki & Kleihues, 2005; Stupp, Mason, & Bent, 2005). GBM incidence and prognosis have changed over the past few years. This has been explained by several risk factors, such as sex, age group, race, ethnicity, year of diagnosis, primary site, and surgical removal of the tumor (Pietschmann et al., 2015; Stummer et al., 2008). It has been found that the overall prognosis of patients with GBM is poor, with a median survival of 14.6 months and a five‐year survival rate of <5% (Ostrom et al., 2013; Stupp et al., 2005). A review of the relevant literature, which included a well‐conducted systematic review (Beyer et al., 2016), provided evidence of an association between survival in cases of glioblastoma and several prognostic factors, including age at diagnosis, sex, race/ethnicity, primary site, and treatment (including surgery). However, no information was available about the effect of subtypes of glioblastoma and prognosis, particularly in terms of whether survival in cases of giant cell glioblastoma was different from that in cases of other subtypes of glioblastoma multiforme. Kozak and Moody (2009) conducted a study using the Surveillance, Epidemiology, and End Results (SEER) database from 1988–2004, with which they made a comparison between GCG and GBM and found that GCG had a better prognosis. The present study included samples from 1985 to 2014 to discover the difference in prognosis between glioblastoma subtypes after the evolution of treatment modalities over the past few years. Therefore, the current study aimed to find the differences between GBM and GCG regarding mortality and prognosis among adults and elderly patients in the U.S.

## 2 MATERIALS AND METHODS

### 2.1 Study strategy and data source

A historical cohort was assembled using data from the Surveillance, Epidemiology, and End Results (SEER) database in July 2017 (http://www.seer.cancer.gov/)_._ The data were collected via SEER*Stat software from 1985 to 2014. The SEER program was established in 1973 by the U.S. NCI and collects incidences and survival records of patients with malignant tumors from 18 population‐based cancer registries in the U.S. (SEER). The registries represent approximately 28% of the population of the U.S.; registries were selected, in part, for their diverse population subgroups. These surveys have multistage sampling and are considered to be complex, overestimated, and not representative of the entire U.S. population. However, SEER does its own modeling through extrapolation.

### 2.2 Study population

Patients aged younger than 20 years have a lower incidence rate; frequency rapidly increases starting in the fifth decade of life (Furnari et al., 2007). Therefore, the inclusion criteria for the analysis were patients with a confirmed diagnosis of GBM or GCG at age 18 to 65 from the years 1985–2014. The exclusion criteria included insurance, grading, and tumor size, due to a high percentage (over 25%) of missing data in the SEER database. The SEER database included patients’ insurance data from the years 2007 and onwards. Also, in terms of tumor size, 65% of data was missing in the database. However, glioblastoma has no clear grading system, as it is a type of glioma and is considered the most malignant type (type 4). Therefore, grading was also excluded (http://www.brainlife.org/abstract/2017/Mesfin_F170715.pdf).

### 2.3 Study variables

**Figure 1:**
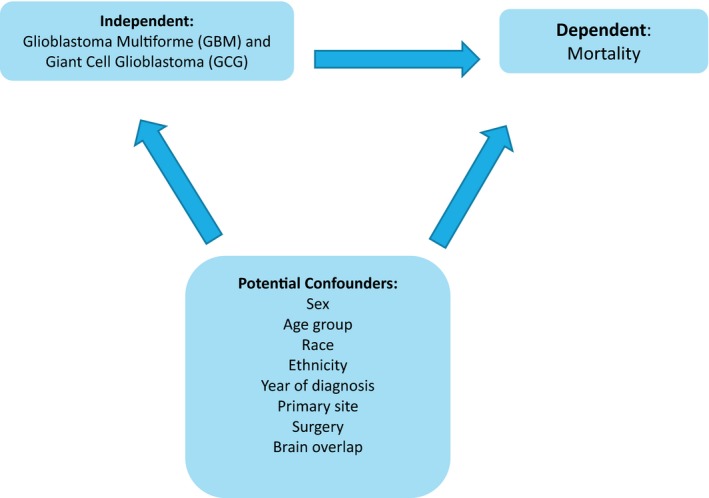
Variables were analyzed using the SEER database and Stata software

The study variables included data of GBM patients (histology codes: ICD‐O‐3:9440/3, 9441/3) with tumors located in several locations: supratentorial (cerebrum, frontal lobe, temporal lobe, parietal lobe, occipital lobe), brain overlap, and infratentorial (cerebellum, ventricle, and brainstem). In addition, primary site codes (C71.0‐C72.0) were extracted from the SEER database. Figure 1 shows the variables that were analyzed.

In addition, the SEER research data record description was used to categorize other variables such as race, which was categorized into White, Black, and Others. Ethnicity was also categorized into Non‐Spanish–Hispanic–Latino and Spanish–Hispanic–Latino. Year of diagnosis was categorized into years before 2010 and years 2010–2014 due to the approval of Bevacizumab for recurrent glioblastoma in 2010 (Johnson, Leeper, & Uhm, 2013).

### 2.4 Statistical analysis

First, the population was selected from the SEER database. Then, the characteristics of the population were described. After that, the general distribution of the data was examined. Next, some variables were transformed into appropriate categories (e.g., group was categorized into adults from 18 to 65 years old and elderly individuals 59–65 years old; Li et al., 2017). The primary site was categorized into supratentorial, brain overlap (including the brain ventricles and other unspecified brain locations), and infratentorial regions.

The alpha level was set at 0.2 due to the small sample size of GCG incidences in the SEER database.

A chi‐square test was used for categorical data. Categorical data were expressed by numbers (*n*) and percentage (%). A univariate analysis was used for variables having a *p*‐value < .05, while potential confounders (patient's sex, age group, race, ethnicity, year of diagnosis, primary site, brain overlap, and surgery) were selected and evaluated by multivariate logistic regression models to calculate the odds ratio with stepwise selection. A collinearity model was used to determine the relationship between each of the confounders for the exclusion of dependent variables. However, no significant relationship between the confounders was excluded.

### 2.5 Ethical considerations

Ethical approval was waived, since the analysis was considered nonhuman subjects research by the Florida International University Health Science Institutional Review Board.

## 3 RESULTS

The study sample was 25,117. It included 24,909 patients with GBM and 208 with GCG. However, 88.3% of patients with GBM died within a few years, while 84.1% of GCG patients also died from the tumor. The baseline characteristics of the study sample are explained in Table 1, which shows that gender has a slight variation in GBM and GCG incidences. Males are more likely to develop GBM than GCG; conversely, females are more likely to develop GCG. Table 1 also shows that the incidence of GBM and GCG is not similar in relation to age group. Hence, it is statistically significant that adults have a higher predisposition to developing GCG than GBM.

**Table 1:** Baseline characteristics of GBM and GCG patients from 1985–2014 in US

Characteristics	Type of glioblastoma		
	GBM NOS *N *(%)	GCG *N *(%)	*p*‐Value
Sex			
Male	14,375 (57.7)	115 (55.3)	.481
Female	10,534 (42.3)	93 (44.7)	
Age group			
Adults (18–59)	10, 221 (41.0)	120 (57.7)	<.001
Elderly (>60)	14,686 (59.0)	88 (42.3)	
Race			
White	22,700 (91.3)	184 (88.5)	.318
Black	1,169 (4.7)	12 (5.8)	
Other	994 (4.0)	12 (5.8)	
Ethnicity			
Non‐Spanish–Hispanic–Latino	23,791 (95.5)	192 (92.3)	.027
Spanish–Hispanic–Latino	1,118 (4.5)	16 (8)	
Year of diagnosis			
Before 2010	20,719 (83.3)	171 (82.2)	.71
2010–2014	4,190 (16.8)	37 (17.8)	
Primary site			
Supratentorial	17,828 (71.6)	168 (80.8)	<.001
Brain overlap	6,767 (27.2)	33 (15.9)	
Infratentorial	314 (1.3)	7 (3.4)	
Surgery			
None	3,287 (26.1)	13 (11.4)	<.001
No GTR	5,719 (45.5)	50 (43.9)	
GTR	3,574 (28.4)	51 (44.7)	

Abbreviations: GBM, glioblastoma multiforme; GCG, giant cell glioblastoma; GTR, gross total resection; NOS, not otherwise specified.

Race also reveals some variations in terms of the two subtypes of glioblastoma, with individuals who have a white racial background being more prone to GBM, while individuals of other races being more prone to GCG. The Non‐Spanish–Hispanic–Latino ethnicity has a slightly higher incidence of GBM than GCG, while, inversely, Spanish–Hispanic–Latinos have fewer incidences of GBM than GCG. The incidence of GBM was slightly higher than the incidence of GCG before 2010; after 2010, the incidence of GCG was higher. However, incidences of both tumors have decreased considerably since 2010.

The study reveals some statistically significant differences in terms of tumor primary site, with high statistical significance. Both subtypes of tumors originate more often in the supratentorial part of the brain than elsewhere in the central nervous system. However, GCG tumors originate more from the supratentorial site than do GBM tumors. It is also statistically significant that GBM risk is higher in patients with no surgery or no gross total resection, while patients with gross total resection (GTR) have an elevated GCG risk. Table 2 shows that patients with GBM have a higher mortality rate than do GCG patients. Table 3 shows that GCG has an odds ratio [OR] of 0.56 with a confidence interval of 0.53–1.44, which is independently associated with reduced mortality.

**Table 2:** Mortality rate of GBM and GCG patients from 1985–2014 in US

Characteristics	Mortality		*p*‐Value
	Alive *N* (%)	Dead *N* (%)	
Glioblastoma			
GBM	2,916 (11.7)	21,993 (88.3)	.064
GC	33 (15.9)	175 (84.1)	
Sex			
Male	1,778 (12.3)	12,703 (87.7)	<.001
Female	1,162 (10.9)	9,465 (89.1)	
Age group			
Adults	1,464 (14.2)	8,877 (85.8)	<.001
Elderly	1,483 (10.0)	13,291 (90.0)	
Race			
White	2,534 (11.1)	20,350 (88.9)	<.001
Black	200 (16.9)	981 (83.1)	
Others	198 (19.7)	808 (80.3)	
Ethnicity			
Non‐Spanish–Hispanic	2,741 (11.4)	21,242 (88.6)	<.001
Spanish–Hispanic–Latino	208 (18.3)	926 (81.7)	

Abbreviations: GBM, glioblastoma multiforme; GCG, giant cell glioblastoma.

**Table 3:** Odds ratio of GBM and GCG patients from 1985–2014 in US

Characteristics	Unadjusted		Adjusted^a^	
	OR (95% CI)	*N*	OR (95% CI)	*N*
Glioblastoma				
GBM	Reference			
GCG	0.70 (0.5–1.02)	25,117	0.88 (0.53–1.44)	12,694

Abbreviations: CI, confidence interval; GBM, glioblastoma multiforme; GCG, giant cell glioblastoma; OR, odds ratio.

a Adjusted for age, sex, race, ethnicity, year of diagnosis, primary site surgery.

Table 2 also shows a slight difference in mortality between age groups in relation to the two glioblastoma subtypes; this difference is statistically significant. It indicates that elderly patients have a worse prognosis than do adults. Glioblastoma patients with a white racial background also face a slightly increased risk of death. The Spanish–Hispanic–Latino ethnicity has a lower mortality rate than do Non‐Spanish–Hispanic–Latinos, as explained in Table 3. The Spanish–Hispanic–Latino ethnicity is independently protective from GBM and GCG (OR 0.63, CI = 0.52–0.77). GBM and GCG tumors with brain overlap have a statistically significant worse outcome than do other primary tumor sites, as shown in Table 2.

Surgery also plays a role in patients’ outcomes. The mortality rate increases in patients with no tumor resection. As shown in Table 3, the factors independently associated with increased mortality are: being female ([OR] 1.12, CI = 1.01–1.25), being age 59 to 65 years (OR 1.64, CI = 1.48–1.82), and being diagnosed earlier than 2010 (OR 5.26, CI = 4.74–5.84). Table 4 shows some secondary findings of the study.

**Table 4:** Secondary findings of race/ethnicity and the year of diagnosis

Characteristics	Unadjusted		Adjusted	
	OR (95% CI)	*p*‐Value	OR (95% CI)	*p*‐Value
Race				
White	REF			
Black	0.61 (0.52–0.71)	<.001	0.64 (0.52–0.79)	<.001
Others	0.50 (0.43–0.60)	<.001	0.61 (0.50–0.75)	<.001
Ethnicity				
Non‐Spanish–Hispanic	REF			
Spanish–Hispanic–Latino	0.57 (0.49–0.67)	<.001	0.63 (0.52–0.77)	<.001
Year of diagnosis				
Before 2010	5.44 (5.01–5.91)	<.001	5.26 (4.74–5.84)	<.001
2010–2014	REF			

Abbreviations: CI, confidence interval; OR, odds ratio.

## 4 DISCUSSION

To the best of our knowledge, this study is one of the few that address the association of subtype of glioblastoma and mortality in adults in the U.S. after 2010 and that involves a large sample size in GCG and GBM with the utilization of ICD‐0‐3 codes. GBM is more common than GCG and has a higher mortality rate. On the other hand, the current study provides statistically significant data about ethnicity, explaining that the Spanish–Hispanic–Latino ethnicity is independently protective from both glioblastoma subtypes as compared to the Non‐Spanish–Hispanic ethnicity. Furthermore, factors like being female, being age 59 to 65, and having a year of diagnosis before 2010 are independently associated with increased mortality.

This study found that elderly individuals have the highest mortality rate among GBM and GCG patients in comparison to adults (*p* < .001). Some studies were consistent with the previous findings (Hartmann et al., 2010; Lin & Wagner, 2015; Murthy, Krumholz, & Gross, 2004; Rong et al., 2016). Therefore, age is considered a significant predictor of survival time (Shah, Bista, & Sharma, 2016). This study also demonstrates that elderly individuals are more prone to having GBM than GCG, which explains the rarity of GCG. This finding may indicate that the elderly population is more susceptible to GBM due to an increased chance that cells will mutate into cancer cells. The current study demonstrated that more males are afflicted with GBM than with GCG, while more females are afflicted with GCG (*p* = .481), consistent with (Colen, Wang, Singh, Gutman, & Zinn, 2015; Matsuda et al., 2011; Nizamutdinov et al., 2018; Ohgaki et al., 2004; Shinojima et al., 2004; Verger et al., 2011). Another study, conducted on Black patients with GBM, showed that Black males were affected by GBM more than were Black females (Loukas, 2014). Therefore, GCG, an uncommon type of glioblastoma multiforme, more often affects females. However, GBM affects males more than females, regardless of race. The previous findings may be explained by genetic factors.

The present study stated that the mortality rate is higher among GBM and GCG patients diagnosed before 2010 (*p* < .001). Also, one study showed that the prognosis for elderly patients with glioblastoma has improved since the introduction of the Stupp regimen (i.e., radiotherapy plus concomitant and adjuvant temozolomide) in 2005 (Shah et al., 2016). This indicates that year of diagnosis has a significant impact on the prognosis of glioblastoma patients. However, the proportion of patients with GBM is slightly higher than the proportion of GCG patients before 2010. On the other hand, the proportion of GCG incidences is slightly higher than the proportion of GBM incidences after 2010 (*p* = .71).

Patients who did not have a GTR have a higher mortality rate (*p* < .001). Moreover, patients who had not undergone surgery or GTR developed GBM more often than they did GCG (*p* < .001).

Studies like (Koul, Dubey, Torri, Kakumanu, & Goyal, 2012; Pan, Ferguson, & Lam, 2015) had similar findings, stating that GTR has a better survival rate than does partial resection or biopsy. Brain overlap GBM and GCG tumors are associated with higher mortality rates than are supratentorial and infratentorial tumors (*p* < .001). This finding was similar in one study (Becker et al., 1967).

However, another study showed that the median survival time for both cerebellar GBM (cGBM) and supratentorial GBM (sGBM) patients is 8 months, though sGBM had a worse prognosis as the study progressed (Jeswani et al., 2013). Also, patients with brain overlap tumors have a higher tendency to develop GBM than GCG (*p* < .001). Because GBM is more common than GCG, it affects brain overlap regions more than supra‐ and infratentorial regions (which are affected more by GCG, *p* < .001). This accounts for the higher mortality rate. Non‐Spanish–Hispanic people have a higher mortality rate from GBM (88.6%, *p* < .001). In addition, a study done on Americans with glioblastoma suggested that Latinos tend to have a lower incidence of GBM and present slightly younger than non‐Latino Whites (Shabihkhani et al., 2017).

However, white people were found to have the highest incidence of death from GBM and GCG as compared to individuals of other races (*p* < .001).

### 4.1 Limitations

Unfortunately, SEER registry has some unregistered variables, underreported and missing data regarding surgery followed by chemo‐ or radiotherapy. There were also different styles in data coding and reporting, and movement of patients in and out of SEER registry areas.

Furthermore, SEER database may have been affected by the selection bias. In which, prospective studies might be influenced as well. For example, immortal time bias in the assessment of surgery followed by chemo‐ or radiotherapy effectiveness.

## 5 CONCLUSIONS

GCG was not statistically significant in terms of its association with reduced mortality. Factors such as being female, being age 59 to 65, and having a year of diagnosis before 2010 were independently associated with increased mortality. The Spanish–Hispanic ethnicity was independently protective from GBM and GCG as compared to the Non‐Spanish–Hispanic ethnicity. Additional studies should be conducted on GBM and GCG patients with the inclusion of important factors such as tumor size/activity, disease stage, treatment history, and insurance.

## CONFLICTS OF INTEREST

The authors declare no conflict of interest.

## AUTHOR CONTRIBUTIONS

Conceptualization, A.K.B and J.G.R.; methodology, A.K.B; formal analysis, A.K.B; Y.R.B; A.M.F J.G.R and K.A.B; Supervision, K.A.B and J.G.R; writing—original draft preparation, A.K.B; Y.R.B; A.M.F, J.G.R; K.A.B writing—review and editing, A.K.B and K.A.B

## Data Availability

The Surveillance, Epidemiology, and End Results (SEER) data used to support the findings of this study were supplied by the National Cancer Institute under license and so cannot be made freely available. Requests for access to these data should be made to the National Cancer Institute (http://www.seer.cancer.gov/).
